# Effects of Voluntary Running Wheel Exercise-Induced Extracellular Vesicles on Anxiety

**DOI:** 10.3389/fnmol.2021.665800

**Published:** 2021-07-01

**Authors:** Kyeong Jin Yoon, Suhong Park, Seung Hee Kwak, Hyo Youl Moon

**Affiliations:** ^1^Department of Physical Education, Seoul National University, Seoul, South Korea; ^2^Institute of Sport Science, Seoul National University, Seoul, South Korea; ^3^Institute on Aging, Seoul National University, Seoul, South Korea

**Keywords:** exercise, extracellular vesicle, anxiety, miRNA, ICV injection

## Abstract

Anxiety disorders are the most frequently diagnosed psychological condition, associated with serious comorbidities including excessive fear and interference with daily life. Drugs for anxiety disorders are typically prescribed but the side effects include weight gain, nausea, and sleepiness. Exercise is an effective treatment for anxiety. Exercise induces the release of extracellular vesicles (EVs) into the circulation, which transmit signals between organs. However, the effects of exercise-induced EVs on anxiety remain poorly understood. Here, we isolated EVs from the sera of mice that were sedentary or that voluntarily exercised. We characterized the changes in the miRNA profile of serum EVs after 4 weeks of voluntary exercise. miRNA sequencing showed that 82 miRNAs (46 of which were positive and 36 negative regulators) changed after exercise. We selected genes affected by at least two miRNAs. Of these, 27.27% were associated with neurotrophin signaling (9.09% with each of central nervous system neuronal development, cerebral cortical cell migration, and peripheral neuronal development). We also analyzed behavioral changes in mice with 3 weeks of restraint stress-induced anxiety after injection of 20 μg amounts of EVs from exercised or sedentary mice into the left cerebral ventricle. We found that exercise-derived EVs reduced anxiety (compared to a control group) in a nest-building test but found no between-group differences in the rotarod or open field tests. Exercise-derived EVs enhanced the expression of neuroactive ligand-receptor interaction genes. Thus, exercise-derived EVs may exhibit anti-anxiety effects and may be of therapeutic utility.

## Introduction

Anxiety disorders are the most common mental illnesses in the United States; 31% of adults experience anxiety disorders at some time during life (Ritchie and Roser, 2018). Anxiety disorders reduce the quality-of-life (QoL) and increase mortality (Pande et al., 2013; Sarma and Byrne, 2014; Walker et al., 2015). Such disorders are accompanied by depression and other mental illnesses, imposing major societal burdens (Wittchen, 2002). The key anxiety-related brain regions are the amygdala, the ventromedial prefrontal cortex (vmPFC), the anterior cingulate cortex (ACC), and the hippocampus (Milad and Quirk, 2012). Poor coordination among these regions may cause anxiety and fear. Also, a dysfunctional hypothalamic-pituitary-adrenal (HPA) axis may trigger anxiety; the HPA axis becomes hyperactive during stress (Craske et al., 2017). Platelet 5-hydroxytryptamine (5-HT, serotonin)-reuptake-site binding was decreased and brain-derived neurotrophic factor (BDNF) downregulated in patients with anxiety disorders (Iny et al., 1994; Janke et al., 2015).

Typical treatments include selective serotonin reuptake inhibitors (SSRIs), and serotonin and norepinephrine reuptake inhibitors (SNRIs). However, the side effects such as weight gain, nausea, sleepiness (Lord et al., 2017) and anticholinergic effects (Bet et al., 2013). Therefore, other therapies are required. Exercise can be an effective treatment for anxiety disorder. Those who engaged in aerobic exercise and resistance training had lower anxiety symptom scores than controls (Herring et al., 2012). In addition, a meta-analysis found that physical activity positively affected post-traumatic stress disorder (PTSD), which is a type of anxiety disorder (Rosenbaum et al., 2015).

Physical activity plays a key role in human health, affecting protein synthesis and degradation, gene regulation, and epigenetic controls including methylation and acetylation (Widmann et al., 2019; Yoon et al., 2019). Exercise triggers exerkines release into the circulation and changes Extracellular vesicles (EVs) composition (Safdar et al., 2016). EVs contain cellular proteins, nucleic acids, lipids, and metabolites within bilayered membranes. EVs transfer signals among organs (Pitt et al., 2016; Maji et al., 2017). Acute aerobic exercise did not change EVs size but the levels of concentration increased (Oliveira et al., 2018). EVs protein profile changed after a 1-h cycling exercise in healthy humans (Whitham et al., 2018); exercise-derived EVs contained neurogenesis-related proteins including BDNF, apelin (APLN), and cathepsin B (CTSB) (Safdar and Tarnopolsky, 2018). The microRNAs (miRNAs) of rat EVs changed after acute uphill or downhill exercise (Yin et al., 2019).

Among those, the miRNA profiles of EVs are attracting increasing attention. PubMed shows that only five relevant reports appeared in 2010, while 202 were published in 2019. miRNAs are small non-coding RNAs regulating protein expression *via* mRNA degradation or inhibition of mRNA translation. Exercise-induced miRNAs in EVs protect against cardiovascular disease and strengthen the immune system (Bei et al., 2017; Maji et al., 2017). Two weeks of treadmill exercise changed the EVs miRNA profile to favor neural maturation (Moon et al., 2020). However, no study has explored the effects of exercise-induced EVs miRNAs on anxiety, which is the focus of this study.

## Materials and Methods

### Neuro2A Cell Culture

Neuro2A cells (ATCC CCL-131, USA), a mouse neuroblastoma cell line, were seeded into 6-well plates at an initial density of 10^4^ cells/well and grown in Eagle's Minimum Essential Medium (EMEM; Gibco, NY, USA) supplemented with 10% fetal bovine serum (FBS, Gibco) in a 37 °C incubator with 95% air and 5% carbon dioxide (CO_2_). The cells were passaged every second day and confluency was maintained below 90%.

### Cell Viability Assay

Cell viability was assessed using Cell Counting Kit-8 (CCK-8; Dojin Laboratories, Kumamoto, Japan). Briefly, Neuro2A cells were seeded into 96 well plates at an initial density of 10^3^cells/well. After 24 h on EMEM with 10% exosome-depleted fetal bovine serum (FBS, Gibco), the medium was changed to serum free EMEM. After incubation with the FBS or the indicated concentraiont (0.1, 1, 10 ng) of EVs from sedentary or exercise group for the indicated time, reagent was added and the mixture was incubated for 3 h. To assess cell viability, absorbance at 450 nm was measured using a Tecan (Infinite 200 PRO series) 96-well microplate spectrophotometer (Mannedorf, Switzerland).

### Animal Care

All animal experiments were approved by the Institutional Animal Care and Use Committee (IACUC) of Seoul National University (no. IACUC-190730-6-1). Male C57BL/6J mice were housed at 22–25°C under a 12-h:12-h light:dark cycle with free access to water and food. Mice were housed singly for 4 weeks with or without access to a running wheel.

### Restraint Stress

Restraint was performed 6 h per day during 21 days consecutively. Mice were randomly divided into two groups. Controls (*N, n* = 5) were left completely undetained, while restraint (*R, n* = 15) were received restraint stress for 3 weeks. The restrainers were purchased commercially (JEUNG DO BIO&PLANT CO, LTD, JD-R-02.).

### EVs Collection

After 4 weeks of exercise, mice were anesthesized with isoflurane and whole heart blood collected into BD SST Gold tubes (BD Microtainer, #365957) and centrifuged at 4,000 rpm for 10 min at 4°C; the sera were transferred to fresh tubes. An ExoLutE Plasma and Serum Kit (Rosetta Exosome, #EX-03) was used to remove lipoproteins. Sera (0.5-mL amounts) were centrifuged at 2,000 × g for 10 min at 4°C, the supernatants transferred to fresh microcentrifuge tubes, and the debris removed, followed by two centrifugations with the application of liporemoval column each for 1 min at 500 × g and 4°C. A miRCURY Exosome Cell/Urine/CSF Kit (Qiagen, #76743) was used to precipitate EVs. Precipitation buffer B was added to pretreated serum, followed by overnight incubation and centrifugation at 10,000 × g for 30 min at 20°C. The supernatants were discarded.

### Nanoparticle Tracking Analysis (NTA)

All samples were thawed at room temperature and diluted in PBS to final volume of 1 ml. Smaple was introduced to the NanoSight LM10 (NanoSight Ltd., Amesbury, United Kingdom) through glass prism.

Following settings were set acoording to the previous study (Dragovic et al., 2011; Soo et al., 2012). All particles that dispersed enough light to be above the detection threshold are counted by the NTA software version 2.3 and this is used to determine the concentration.

### miRNA Sequencing

The RNA isolated from each sample was used to construct sequencing libraries with the SMARTer smRNA-Seq Kit for Illumina, following the manufacturer's protocol. Briefly, Input RNA is first polyadenylated in order to provide a priming sequence for an oligo-(dT) primer. cDNA synthesis is primed by the 3′ smRNA dT Primer, which incorporates an adapter sequence at the 5′ end of each RNA template, it adds non templated nucleotides which are bound by the SMRT smRNA Oligo-enhanced with locked nucleic acid (LNA) technology for greater sensitivity. In the template-switching step, PrimeScript RT uses the SMART smRNA Oligo as a template for the addition of a second adapter sequence to the 3′ end of each first-strand cDNA molecule. In the next step, full-length Illumina adapters (including index sequences for sample multiplexing) are added during PCR amplification. The Forward PCR Primer binds to the sequence added by the SMART smRNA Oligo, while the Reverse PCR Primer binds to the sequence added by the 3' smRNA dT Primer. Resulting library cDNA molecules include sequences required for clustering on an Illumina flow cell.

The libraries were validated by checking the size, purity, and concentration on the Agilent Bioanalyzer. The libraries were pooled in equimolar amounts, and sequenced on an Illumina HiSeq 2500 instrument to generate 51 base reads. Image decomposition and quality values calculation were performed using the modules of the Illumina pipeline.

### Intracerebroventricular (ICV) Injection

Mice were anesthetized with isoflurane and placed in a stereotactic frame. The lateral ventricle(AP: −0.5 mm, ML: 1 mm, DV: −2.5 mm) was defined using the bregma and lambda values and 20 μg of EVs injected.

### Tissue Collection

Tissues were collected the day after the 4 weeks of exercise and 3 days after ICV injection. Mice were anesthetized with isoflurane and whole heart blood collected. After perfusion with phosphate-buffered saline, whole brains were collected and samples placed in 4% (v/v) paraformaldehyde or frozen at −80°C.

### Biochemical Tests

(i) Reverse transcription-quantitative polymerase chain reaction (RT-qPCR)

Total RNA was isolated and purified using TRIzol reagent (Invitrogen, #15596026) according to the manufacturer's protocol. cDNA was prepared to employ a CycleScript RT premix (Bioneer, #K-2044-CFG). All primer sequences are listed in Table 1. Expression levels were calculated using a SensiFAST SYBR Lo-ROX Kit (Bioline, #BIO-94020) and a commercial detection system (BioRad, #CFX96).

**Table 1:** RT-qPCR primers list.

**Gene name**	**Sequence (5^**′**^ → 3^**′**^)**
Ndufa	F: CCGGGGTGTCCACTGCGTACA
	R: CGCGTTCCATCAGATACCACTGGT
Sdhb	F: AACATCAACGGAGGCAATAC
	R: CTGGGACTCATCCTTCTTCT
Cox5b	F: GATGAGGAGCAGGCTATGG
	R: GTCTTCCTTGGTGCCTGAAG
Pgc1α	F: AGCCGTGACCACTGACAACGAG
	R: GCTGCATGGTTCTGAGTGCTAAG
Tbp	F: ACCCTTCACCAATGACTCCTATG
	R: ATGATGACTGCAGCAAATCGC

(ii) Western blotting

Total proteins were extracted into RIPA buffer (Thermo Fisher Scientific, #89900) containing a phosphatase inhibitor (Sigma-Aldrich, #4906845001) and a protease inhibitor (Roche, #43693159001), subjected to 10–16% (w/v) Tris-glycine SDS-PAGE, transferred to PVDF membranes using Iblot 2 NC ministacks (Invitrogen, #IB23002), and the membranes blocked with 5% (w/v) skim milk. The primary antibodies were anti-pAMPK (Cell Signaling Technology, #2531S), anti-AMPK (Cell Signaling Technology, #5831S), anti-pACC (Cell Signaling Technology, #3661S), anti-ACC (Cell Signaling Technology, #3662S), and anti-GAPDH (Cell Signaling Technology, #2118S) diluted 1:1000 (primary antibodies) or 1:5000 (secondary antibodies) in TBST (Biosesang, #HT2007) containing 5% (w/v) skim milk. The membranes were then incubated with a peroxidase-conjugated anti-rabbit secondary antibody and signals quantitated using the Immobilion Western Chemiluminescent HRP Substrate (Millipore, #WBKLS0500).

### Behavioral Tests

All behavioral tests were blindly scored by three individuals.

(i) Nest building test

Nest-building behavior commenced at 7:00 pm. Pressed cotton squares (~3 g) were placed in all cages. After dark, every cage was photographed and the cotton removed. Nest-building was scored using the Deacon method (Deacon, 2006).

(ii) Open field test

The open-field test square was a 40 × 40 cm black, polyvinyl chloride, plastic board surrounded by a 40-cm wall. Areas within 10 cm of the wall were considered to be peripheral and other areas central. Time spent in the center and the number of center entries increase when anxiety is low. At the beginning of the test, mice were placed in the central area and were allowed to move freely for 5 min. All sessions were recorded by a video camera placed 145 cm above the square and the data analyzed with the aid of the ANY-maze Video Tracking System (Stoelting Co., Wood Dale, IL, USA). The software detects an animal-based on its contrast with the background. After every session, the square was cleaned with a paper towel dampened with 70% (v/v) alcohol. All open field tests were conducted at 3:00 PM.

(iii) Tail suspension test

The tail suspension test was performed using the method of (Can et al., 2012). Each mouse was suspended on a climbstopper for 6 min by applying adhesive tape to the tail. The climbstopper disturbs the tail-climbing habit of the C57BL/6J mouse. The total duration of immobility was measured in seconds. Mobility was defined as any bodily shaking or limb movement akin to running. Immobility was defined as the absence of movement apart from breathing motion.

(iv) Forced swim test

The forced swim test was rehearsed on the day before the test. Each mouse was placed into a 2-L beaker containing a 20-cm depth of water at 23–25°C for 5 min; and immobility, and swimming and climbing behavior, was scored every 5 s.

### Microarray and Bioinformatic Analysis

Each hippocampus from non-restrained vehicle-injected group (NV, *n* = 3), restraint-stressed sedentary EV-injected group (RS, *n* = 4), restraint-stressed exercise EV-injected group (RE, *n* = 4) were pooled and RNA purity and integrity were confirmed by ND-1000 Spectrophotometer (Nano-Drop, Wilmington, USA). The Affymetrix Whole transcript Expression array process was performed according to the protocol of the manufacturer (Gene Chip Whole Transcript PLUS reagent Kit). cDNA was synthesized according to the manufacturer's method using Gene Chip Whole Transcript (WT) Amplification kit, and the hybridized array was scanned using a GCS3000 Scanner (Affymetrix). Signal values were calculated using Affymetrix® Gene ChipTM Command Console software. Gene enrichment and functional annotation analysis were performed on genes showing significant expression level (≥2-fold) based on gene ontology and KEGG pathway analysis, respectively.

### Statistical Analysis

Statistical analysis was performed with the aid of Graph Pad Prism ver. 7 (Graph Pad Software Inc., La Jolla, CA, USA); all data are presented as means with SEMs. A *p*-value < 0.05 was considered to reflect statistical significance. Student's *t*-test was used to compare data between the two groups.

## Results

### Effects of 4 Weeks of Voluntary Wheel Exercise on Skeletal Muscle Exercise-Related AMPK Signaling Protein and Oxidative Phosphorylated mRNA Levels

**Figure 1:**
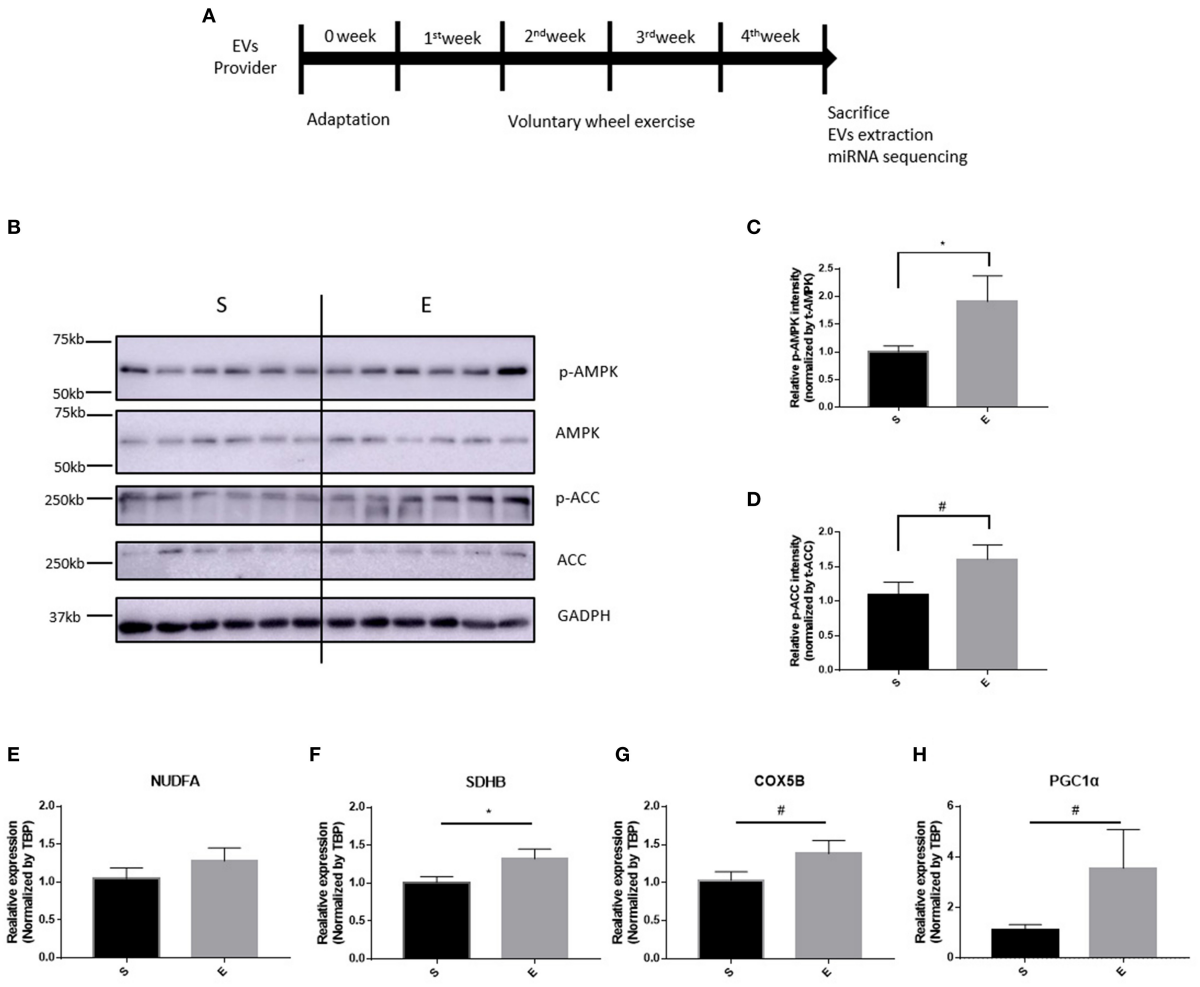
The effects of 4 weeks of voluntary wheel exercise on skeletal muscle proteins and their encoding mRNA levels. **(A)** The experimental schedule. **(B–D)** p-AMPK/AMPK and p-ACC/ACC protein levels. **(E–H)** OXPHOS and Pgc1α mRNA expression levels in the gastrocnemius. *p < 0.05 vs. S. Statistical analysis was performed with the aid of the one-tailed Student *t*-test. EVs, Extracellular vesicles; S, sedentary group (*n* = 6); E, exercise group (*n* = 6). The data represent means ± SEM (*p < 0.05). #*p* < 0.05.

To validate the effects of 4 weeks of voluntary wheel exercise, mice were individually housed with or without an exercise wheel for 4 weeks (Figure 1A) and the levels of exercise-related proteins (phosphorylated AMPK and ACC) measured, as were the levels of encoding mRNAs (oxidative phosphorylation [Nudfa, Sdhb, and Cox5b], and Pgc1α). AMPK and ACC are an important signaling molecule that is activated during exercise training (Chen et al., 2003). The p-AMPK/AMPK and p-ACC/ACC ratios of exercise group was significantly higher than sedentary group (Figures 1B–D, p-AMPK/AMPK, *p* < 0.05 and p-ACC/ACC, *p* = 0.0507). Endurance exercises upregulates the gene expressions of oxidative phosphorylation and Pgc1α that assist promote mitochondrial biogenesis in skeletal muscles (Gureev et al., 2019). Nudfa expression levels did not differ between the sedentary and exercise groups. Sdhb expression was upregulated in the exercise group (*p* < 0.05) and the levels of Cox5b and Pgc1α tended (*p* = 0.06, *p* = 0.07, respectively) to increase in that group (Figures 1E–H). These experiments indicated that 4 weeks of voluntary wheel exercise was sufficient for further study.

### 4 Weeks of Running Wheel Exercise Did Not Changed Serum-Derived EVs External Properties: Size and Number

**Figure 2:**
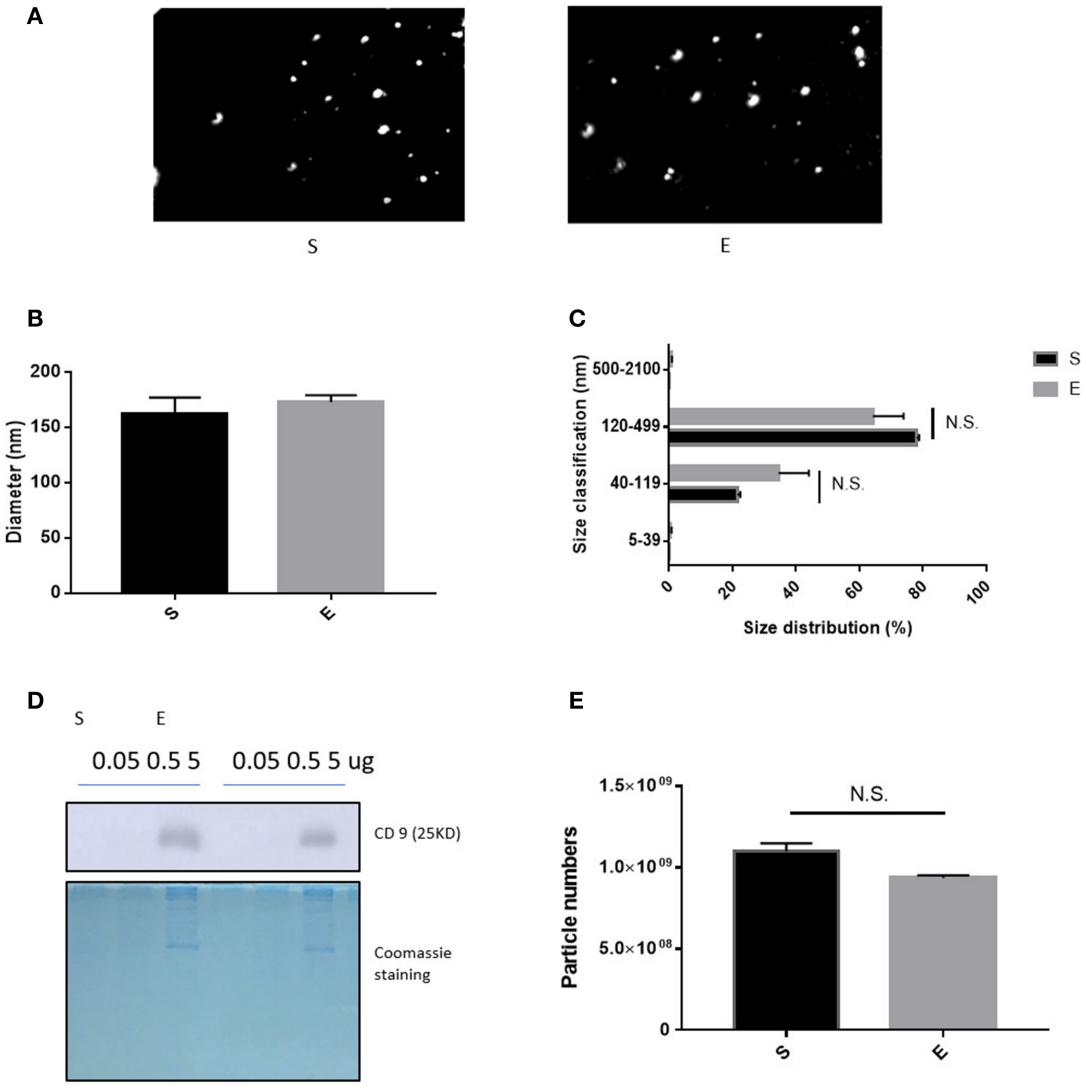
The features of EVs induced by 4 weeks of voluntary wheel exercise. **(A)** A representative video frame. **(B)** The average diameter. **(C)** The size distribution. **(D)** Western blotting for CD9 (an EV-specific marker). **(E)** EV particle numbers. N.S., not significant. Statistical analysis was performed with the aid of the two-tailed Student *t*-test.

After isolation of extracellular vesicles from each group's sera, we performed a nanoparticle tracking assay for investigating the difference between groups; Figure 2A shows the sample frame. Neither EVs diameter nor size changed over the 4 weeks of exercise (Figures 2B,C,E, *p* > 0.05). Figure 2D shows that the level of CD9 (an EVs biochemical marker) was 5 μg/EVs in both groups.

### EVs miRNA Internal Profiles Were Altered After 4 Weeks of Voluntary Wheel Exercise

To figure out the internal composition of extracellular vesicles, we designed the study comparing sedentary and exercise group's miRNA. The EVs of mice that engaged in 4 weeks of voluntary wheel exercise exhibited 46 upregulated and 36 downregulated miRNAs compared to sedentary group (Tables 2A,B). Particularly, we confirmed 4 miRNAs to validate the miRNA sequencing (Supplementary Figures 1A–D). All changed miRNAs were functionally annotated by DAVID ver. 6.7 software, except those lacking ENTERZ GENE IDs. The miRNAs affected the micro-ribonucleoprotein complex, cancer development, the responses to oxygen and amino acids, and gene silencing (Table 3).

**Table 2A:** The list of upregulated miRNAs in 4 weeks of voluntary wheel exercise.

**Mature_ID**	**Exe/Sed.fc**	***p*-value**	**Mature_ID**	**Exe/Sed.fc**	***p*-value**
mmu-miR-215-5p	18.585596	2.96E-41	mmu-miR-429-3p	3.860804	0.000349157
mmu-miR-183-5p	15.017577	7.19E-06	mmu-miR-574-5p	3.800425	8.80E-11
mmu-miR-297a-3p	11.397447	0.018516041	mmu-let-7b-3p	3.799884	7.03E-10
mmu-miR-297b-3p	11.397447	0.018516041	mmu-miR-423-5p	3.78057	6.17E-15
mmu-miR-297c-3p	11.397447	0.018516041	mmu-miR-674-3p	3.130525	0.000129727
mmu-miR-409-3p	10.457163	0.031702174	mmu-let-7e-5p	3.106166	1.05E-08
mmu-miR-511-5p	9.878446	5.24E-05	mmu-let-7k	3.06013	3.17E-06
mmu-miR-1957a	9.539228	8.22E-05	mmu-let-7b-5p	2.957388	3.62E-12
mmu-miR-1306-5p	5.807837	0.0170455	mmu-miR-214-3p	2.725924	5.24E-06
mmu-miR-669c-5p	5.760639	2.95E-05	mmu-let-7f-5p	2.594758	1.19E-09
mmu-miR-10b-5p	5.502284	6.13E-13	mmu-miR-342-3p	2.511935	5.75E-08
mmu-miR-134-5p	5.429372	0.005500209	mmu-let-7a-5p	2.382572	3.55E-08
mmu-miR-205-5p	5.398138	1.78E-08	mmu-miR-151-3p	2.379779	1.24E-05
mmu-miR-365-3p	5.079939	0.000474557	mmu-let-7c-5p	2.36524	5.57E-08
mmu-miR-203-3p	5.075422	1.58E-12	mmu-miR-23a-3p	2.335837	6.16E-08
mmu-miR-29b-3p	4.607671	1.20E-18	mmu-miR-320-3p	2.300079	0.040243773
mmu-miR-574-3p	4.431227	1.34E-19	mmu-miR-206-3p	2.221214	0.000827822
mmu-let-7d-3p	4.391118	2.52E-20	mmu-miR-10a-5p	2.20128	0.00038266
mmu-miR-877-5p	4.385831	4.84E-05	mmu-miR-126a-5p	2.200657	9.78E-07
mmu-miR-466a-3p	4.370751	0.012307727	mmu-miR-140-3p	2.195691	0.001630066
mmu-miR-466b-3p	4.370751	0.012307727	mmu-let-7f-1-3p	2.000093	0.002660807
mmu-miR-466c-3p	4.370751	0.012307727			
mmu-miR-466e-3p	4.370751	0.012307727			
mmu-miR-466p-3p	4.370751	0.012307727			
mmu-miR-5113	4.350275	0.006227222			

**Table 2B:** The list of downregulated miRNAs in 4 weeks of voluntary wheel exercise.

**Mature_ID**	**Exe/Sed.fc**	***p*-value**	**Mature_ID**	**Exe/Sed.fc**	***p*-value**
mmu-miR-30c-5p	−2.113934	0.002034176	mmu-miR-19b-3p	−6.216375	3.14E-14
mmu-miR-1895	−2.129836	0.011726866	mmu-miR-3473b	−7.126822	2.45E-23
mmu-let-7i-5p	−2.144995	2.10E-05	mmu-miR-22-5p	−7.585649	0.000788157
mmu-miR-16-5p	−2.151024	6.78E-05	mmu-miR-128-1-5p	−7.667895	1.20E-18
mmu-miR-130a-3p	−2.183096	0.002372773	mmu-miR-3473e	−8.730115	5.81E-26
mmu-let-7g-5p	−2.450195	5.91E-08	mmu-miR-369-3p	−8.940734	0.001421879
mmu-miR-5128	−2.61082	0.025674902	mmu-miR-148a-3p	−9.930079	7.92E-12
mmu-miR-27b-3p	−2.630326	0.002034176	mmu-miR-322-5p	−10.604367	8.26E-06
mmu-miR-145a-3p	−2.819073	0.000264305	mmu-miR-20a-5p	−11.032222	0.011842362
mmu-miR-133a-3p	−2.886136	0.011147986	mmu-miR-144-5p	−13.387069	8.94E-30
mmu-miR-15b-3p	−2.891632	6.92E-07	mmu-miR-30a-5p	−16.914407	0.000320538
mmu-miR-16-2-3p	−3.008991	0.022498723			
mmu-miR-27a-3p	−3.02509	1.59E-06			
mmu-miR-26a-5p	−3.145651	0.006196773			
mmu-miR-6236	−3.52549	0.004615199			
mmu-miR-451a	−3.626993	1.04E-09			
mmu-miR-128-3p	−3.726309	2.77E-09			
mmu-miR-26b-5p	−4.255282	0.012307727			
mmu-miR-15b-5p	−4.294289	0.000163147			
mmu-miR-99b-5p	−4.494434	5.24E-06			
mmu-miR-194-5p	−4.636119	4.95E-10			
mmu-miR-335-5p	−4.74166	7.60E-07			
mmu-miR-709	−5.177175	0.000161092			
mmu-miR-22-3p	−5.195789	6.51E-12			
mmu-miR-19a-3p	−5.710454	3.59E-11			

**Table 3:** The list of changed miRNA functional annotation.

**Term**	**Count**	**%**	***P*-value**
Micro-ribonucleoprotein complex	27	38.0282	1.80E-57
MicroRNAs in cancer	39	54.9296	9.27E-57
Response to oxygen levels	17	23.9437	5.06E-30
Cellular response to amino acid stimulus	21	29.5775	1.49E-29
Gene silencing by miRNA	14	19.7183	4.09E-22
Cellular response to inorganic substance	13	18.3099	1.17E-20
Cellular response to lipopolysaccharide	14	19.7183	2.53E-14
Cellular response to estrogen stimulus	8	11.2676	1.19E-11
Embryo implantation	6	8.4507	8.62E-07
BMP signaling pathway	6	8.4507	5.49E-06

**Figure 3:**
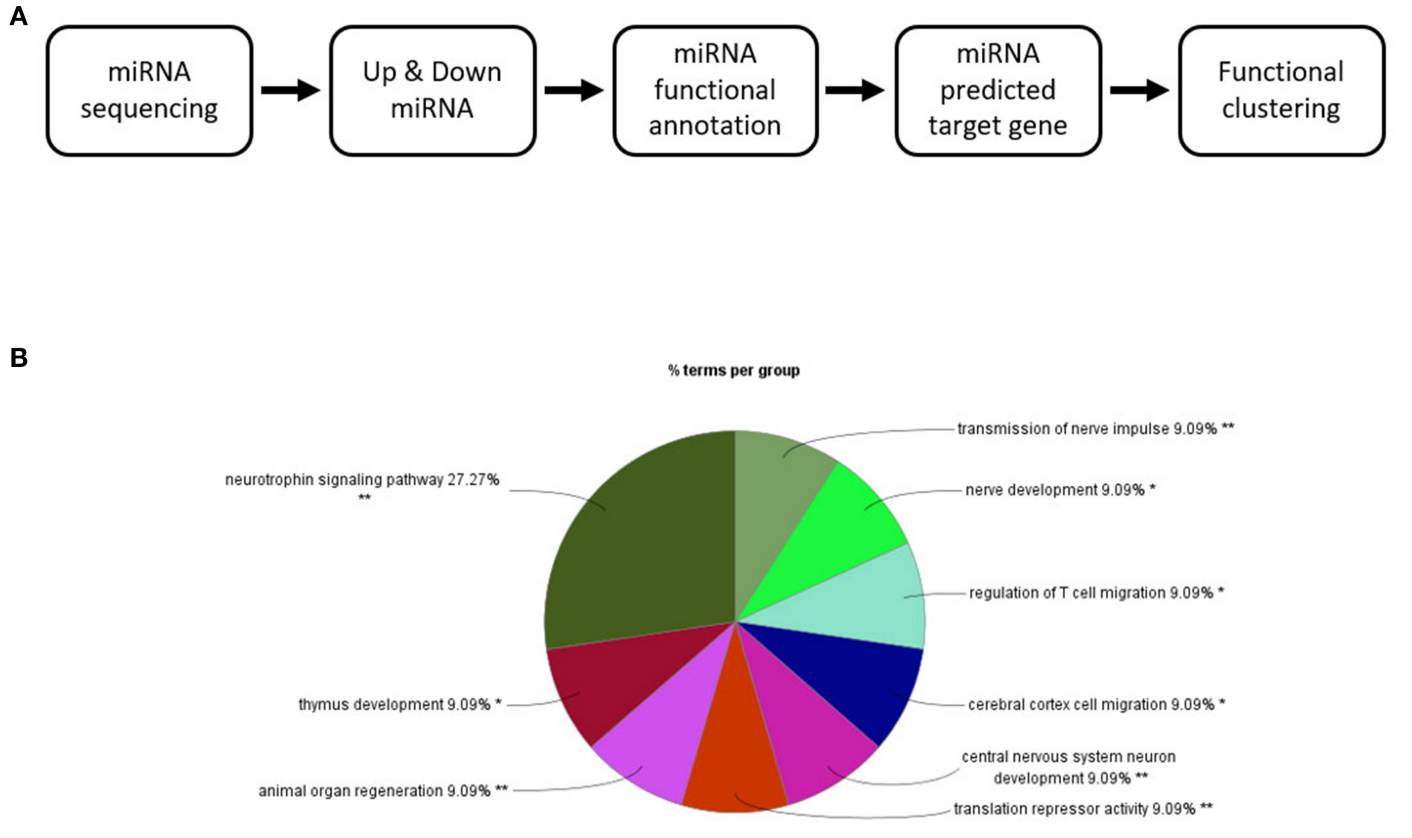
Changes in the EV miRNA profiles after 4 weeks of voluntary wheel exercise. **(A)** A bioinformatic schematic. **(B)** The target genes predicted by functional clustering. Each sedentary, exercise group animal number was 6.

The 82 miRNAs' predicted target genes were identified with the aid of miRNA databases, Target Scan and the miRDB. Both databases identified 129 and 119 genes regulated by more than two miRNAs (Table 4); 13 genes were common to both the Target Scan and miRDB lists. Target scan genes were subjected to functional analysis using Cytoscape (Figure 3A). Of the 129 genes, 27.27% were associated with the neurotrophin signaling pathway (9.09% with each of central nervous system neuronal development, cerebral cortex cell migration, and peripheral neuron development; Figure 3B). These data implied exercise-induced EVs can affect the neuronal cell.

**Table 4:** The list of altered miRNAs predicted target genes.

**Target scan**					
Pkp4	Oas3	Mknk2	Crk	Lifr	Stim2
Unc13b	Nanos1	Adora2b	Klf11	Mdn1	Adcy1
Baz1b	Slc41a1	Ube2g1	Lpar2	Plekha5	Entpd7
Morc3	Hmgxb4	Zfp266	Tfdp1	Map2	Dpysl5
Mtf2	Ltbp2	Usp15	Tbk1	Usp45	E130309F12Rik
Cand1	Ddit4	Epb4.1l3	Pitpnb	Foxg1	Slc5a3
Golgb1	Rnaset2b	Pygo1	Olfr658	Mtmr12	Bach2
4930571K23Rik	Purb	Suz12	Ss18l1	Gpd2	Slc6a19
Egfr	Mgat5	Rnf167	Pnkd	Cep85l	Ccpg1
Map2k6	Mxd1	Rap1a	Casp8ap2	Dmxl1	Rtp1
Bdnf	Defb4	Igsf3	Ick	Cxcl5	Ssh2
Hoxa3	Gm10302	Mylip	Ccnt1	Six6	Hmga2
Srgap1	Stag2	Mpeg1	Ddx6	Slc5a7	Nr6a1
Cntnap5c	Prcp	Aspg	Sfmbt2	Cenpi	Trim71
Gpr64	Limch1	Hs6st1	Zfp113	Zc3h12b	Arid3b
Raver2	Sypl	Aak1	Tmem26	Lmtk2	Pald1
Brinp3	Rqcd1	Vldlr	Psd2	Slc19a2	Sec16b
Pfkfb2	Ttll7	Ppp2ca	Snrk	Traf3	Cacng4
Spn	Meox2	Foxn2	Atp1b4	Pgr	AI118078
Ccdc169	Tmem194b	Fzd7	Pafah1b1	Etl4	Lcor
Zfp275	Plag1	Kcna2	Hif1an	Gm11007	Gm2007
Rgs8	Onecut2	Tet3			
**miRDB**					
Nufip2	Cask	Celsr3	Casz1	Six6	Itga2
Lrp1	Lhfp	Mtdh	Fam98a	Tet2	Tubgcp5
Patl1	Gpm6a	Cyp24a1	Trp53inp1	Ythdf1	Fbxl17
Trim2	Ric1	Rfx6	Atxn1	Adrb3	Alg6
E2f7	Fyco1	Cpeb2	Tnrc6b	Gatm	Unk
Ebf2	Shank1	Syde2	Trim33	Igf2bp1	Dcun1d4
Creb1	Tut4	Cdk19	Taok1	Luzp1	Hic2
Tox4	F3	Adora2b	Kcna4	Twf1	Cxcl5
Rb1cc1	Btaf1	Apaf1	Zmynd11	Rora	Olfr658
Smap1	Hmgxb4	Pds5b	Ubl3	Tnrc6a	Pbx3
Mdga2	Arid4b	Slc26a2	Rapgef2	Pappa	Pfkfb2
Spopl	Pum2	Nrk	Gpr155	Ubfd1	Ino80d
Ppp4r4	Casp8ap2	Ing5	St6galnac6	Plxna4	Fign
Csnk1g3	Ick	Trappc8	Psd2	Akt3	Lin28b
Lrrtm2	Ugt8a	Slc25a25	Mnt	Mapkap1	Stard13
Pes1	Ago2	Erlec1	Rspry1	Rasgef1b	Arid3a
Mei4	Lhx8	Stradb	Rere	Arl2	Trim71
D1Ertd622e	Mkrn3	Greb1l	Hdlbp	Adcy1	Trim6
Ccdc169	Klhl20	Rap2c	Ptprf	Plk2	Nr6a1
Mis18a	Pip4k2a	Tet1	Scn2a	Arhgap6	

### EVs Derived by 4 Weeks of Voluntary Wheel Exercise Increased Neuro2A Cell Viability

**Figure 4:**
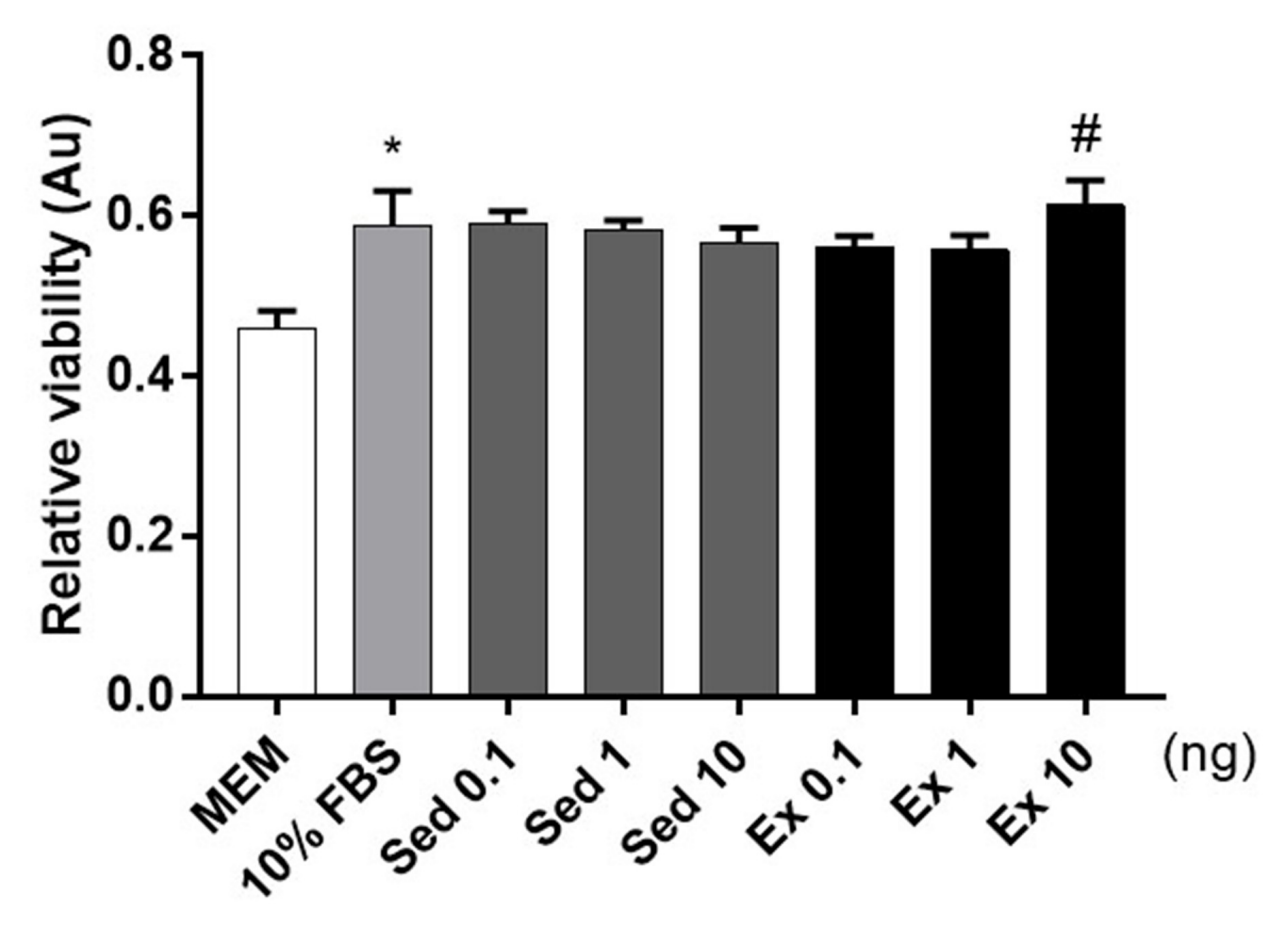
Effects of 4-week, voluntary running wheel exercise-induced EVs on Neuro2A cell viability levels. After 24 h of treatment with MEM alone, 10% (v/v) FBS, or 0.1, 1, or 10 ng of EVs from the sedentary or exercised group. *p < 0.05 compared to the MEM control. ^#^*p* < 0.05 compared to the Sed 10 group. Statistical analysis was performed with the aid of the ANOVA test.

To verify the hypothesis, we conducted cellular viability experiments using Neuro2A, a neuroblastoma cell line. The addition of fetal bovine serum to 10% (v/v) increased Neuro2A cell viability 1.2-fold relative to the MEM-only control (Figure 4). Twenty-four hours treatment of 10 ng of exercise-induced EVs enhanced the viability of Neuro2A cell compared to the same dosage of sedentary (Figure 4). This result that exercise-derived EVs affect positively on Neuro2A cell viability.

### EVs Induced by Running Exercise Ameliorated Anxiety Behaviors

**Figure 5:**
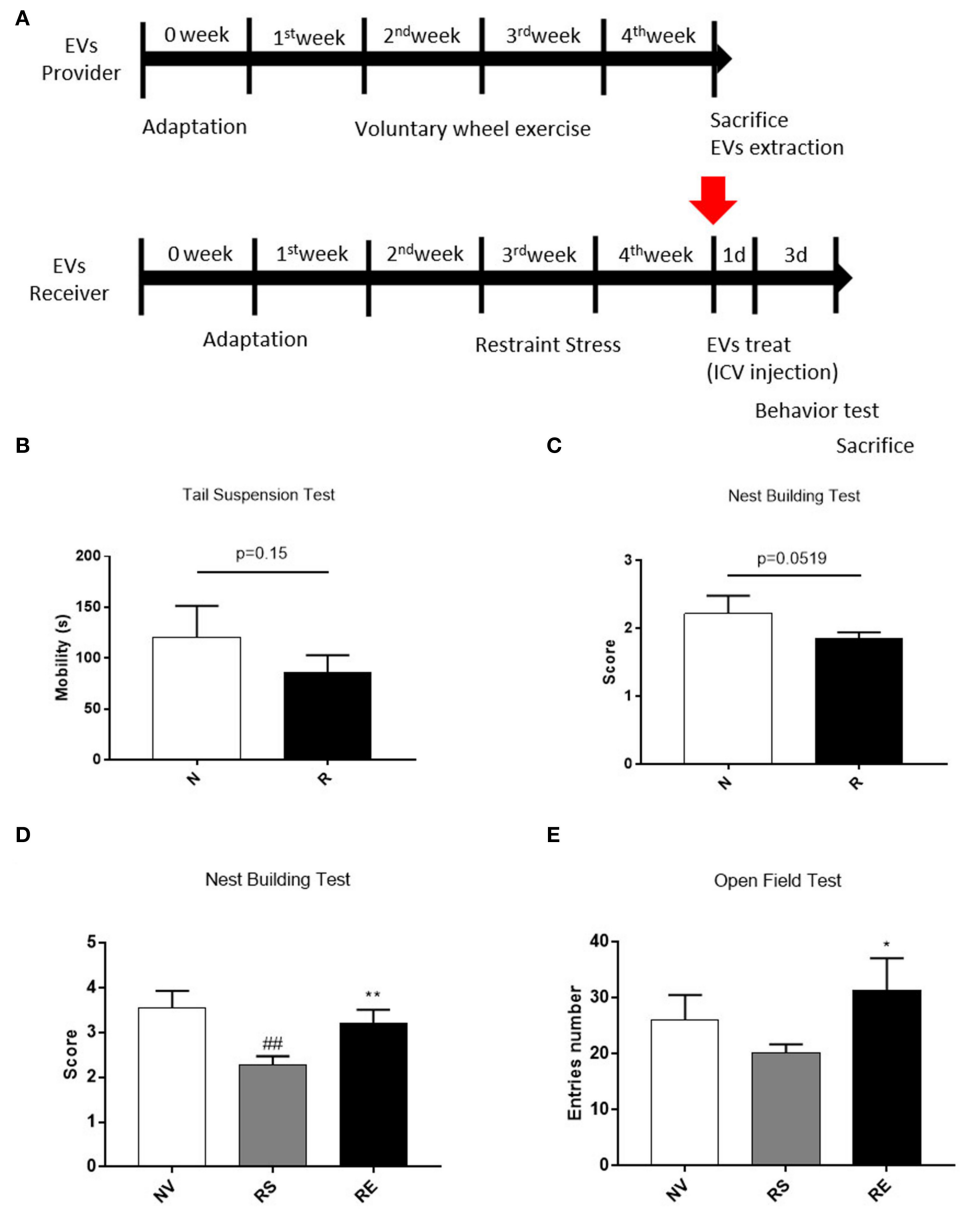
The effects of 4 weeks of voluntary running wheel exercise-induced EVs on anxiety. **(A)** The experimental design (red arrows indicate EV ICV injection). **(B)** The mobility durations of the non-restrained and restrained groups on the tail-suspension test. **(C)** The group scores on the nest- building test prior to ICV injection. **(D)** The group scores on the nest-building test after ICV injection. **(E)** The center entry numbers in the open field test after ICV injection. All mice were male. *N*, non-restrained (6-week-old, *n* = 5); *R*, restrained (thus stressed) (6-week-old, *n* = 15); NV, non-restrained vehicle-injected group (11-week-old, *n* = 5); RS, restraint-stressed sedentary EV-injected group (11-week-old, *n* = 5); RE, restraint-stressed exercise EV-injected group (11-week-old, *n* = 5). *p < 0.05, **p < 0.001 compared to the NV group, ^##^*p* < 0.001 compared to the RS group. Statistical analysis was performed using the one-tailed Student *t*-test.

It is well-known that emotionally afflicted mice are accompanied by decreased neuronal cell viability. Exercise, on the contrary, promotes the production of nerve cells and increases resistance to stress. Therefore, we conducted a behavioral experiment to find out whether exercise-derived EV directly affects beneficial changes in stressed mice. Mice were housed for 4 weeks with or without a wheel after 1 week of acclimatization and restraint stressed mice received EVs from exercised mice *via* ICV injection (Figure 5A). Three weeks of restraint stress-induced a tendency of depression, as revealed by reduced mobility on the tail suspension test (Figure 5B, *p* = 0.15). Restraint stress-treated mice also scored lower than the non-treated group in the nest-building test, thus exhibiting anxiety-like behavior (Figure 5C, *p* = 0.0519).

Exercise-induced EVs injection increased the nest-building test scores compared to sedentary-induced EVs injected (Figure 5D, *p* < 0.001). The number of center entries in the open field test was higher in exercise-induced EVs injected mice (Figure 5E, *p* < 0.05). However, basic behavior did not differ between the two groups (data not shown). These results elucidated that the exercise-derived EVs have the positive effect on anxiety behaviors.

### Restraint Stress and Exercise-Induced EVs Altered Hippocampal mRNA Levels

**Figure 6:**
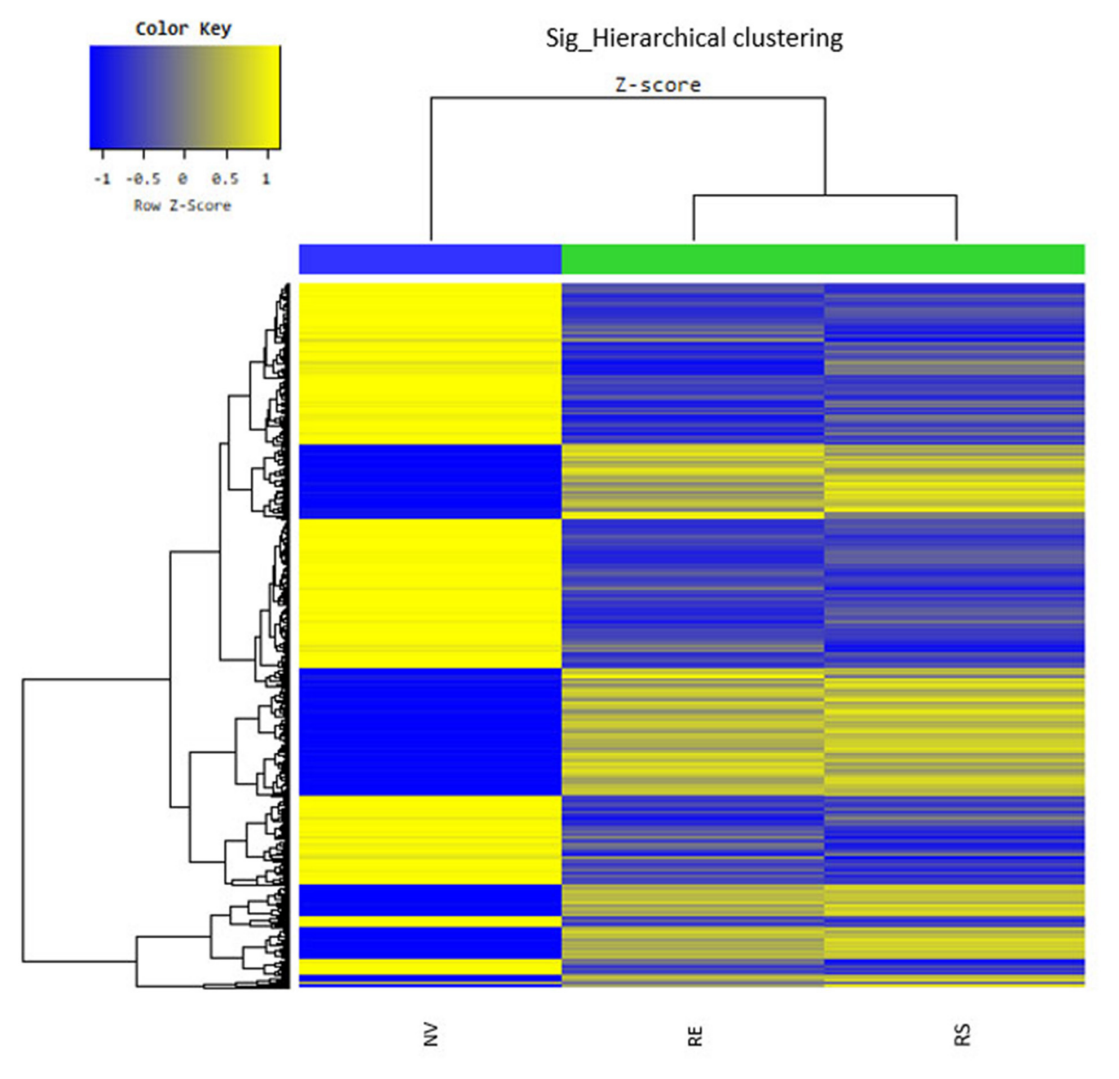
The hippocampal effects of 4 weeks of voluntary wheel exercise-induced EVs. The microarray hierarchical clustering heat-map image. NV, non-restrained vehicle-injected group (*n* = 3); RS, restraint-stressed sedentary EV-injected group (*n* = 4); RE, restraint-stressed exercise EV-injected group (*n* = 4).

Through microarray experiments, we directly investigated changes of gene profiles in the hippocampus of EVs injected mice. Restraint stress transformed the microarray hierarchical clustering heat map image (Figure 6). However, few changes were noted after EVs injection (Figure 6). Restraint stress significantly changed the expression levels of genes associated with long-term depression (Plcb4, Gucy1a3, Prkg2, Ppp1r17, Grid2, and Crhr1) in the Kyoto Encyclopedia of Genes and Genomes (KEGG) map (Table 5). Exercise-induced EVs injection upregulated the expression of genes associated with neuroactive ligand-receptor interaction (Tacr3, Gabrr1, Cysltr1, Oprm1, and Trhr) in the KEGG map (Table 6).

**Table 5:** The list of chaged gene by restraint stress, fold change means restraint/non-restraint.

**KEGG map name**	***p*-value**	**Gene**	**Log2 fold change (R/N)**
Long-term depression	0.01292	Plcb4	−4.791442
		Gucy1a3	−2.459807
		Prkg2	−4.137201
		Ppp1r17	−2.019965
		Grid2	−2.287125
		Crhr1	−2.482984

**Table 6:** The list of changed gene by exercise-induced EVs, fold change means restraint exercise/restraint sedentary.

**KEGG map name**	***p*-value**	**Gene**	**Log2 fold change (RE/RS)**
Neuroactive ligand- receptor interaction	0.000994	Tacr3	2.9314
		Gabrr1	2.529145
		Cysltr1	2.070185
		Oprm1	2.551826
		Trhr	2.312414

## Discussion

Anxiety disorders are characterized by excessive worry about events or activities that persists for at least 6 months (American Psychiatric Association, 2013). Large-scale surveys have found that 33.7% of subjects suffer anxiety disorders during life (Alonso and Lépine, 2007). Anxiety disorders are comorbid with other mental illnesses, creating major societal burdens. However, the common drugs trigger side effects including nausea, weight gain, and sleepiness (Lord et al., 2017). Thus, new therapies are required (Wegner et al., 2014; Stonerock et al., 2015).

Exercise is a powerful treatment, improving PTSD, sub-element anxiety disorders, and generalized anxiety disorder (Herring et al., 2012; Rosenbaum et al., 2015). Exercise increased neural precursor cell proliferation in the mouse dentate gyrus (Pons-Espinal et al., 2019). Besides, exercise intervention may ameliorate not only cognitive but also behavior function in individuals with Parkinson's disease. Physical activity can protect from cognitive impairment in Alzheimer's disease. These experiments attest that exercise helps individuals to mitigate neural disease (Petzinger et al., 2013; Pin-Barre and Laurin, 2015; Cui et al., 2018).

EVs may modulate the beneficial effects of exercise (Safdar and Tarnopolsky, 2018; Whitham et al., 2018). EVs contain proteins, nucleic acids, lipids, and cell metabolites (Maji et al., 2017); and engage in inter-organ signaling (Pitt et al., 2016). Previous studies showed that exercise-induced EVs exerted positive effects on neuronal systems (Safdar and Tarnopolsky, 2018; Moon et al., 2020). However, whether such EVs reduce anxiety remains unclear. Therefore, we designed the experiments and found that anxiety was relieved through intracerebroventricularly injection of such EVs.

Most previous studies found that 4 weeks of voluntary wheel exercise significantly increased exercise-related signaling (Tanner et al., 2013; Kurauti et al., 2016; Liao et al., 2017). Phosphorylated AMPK and ACC, which are activated by exercise, promote ATP production and increase rates of myocardial fatty acid oxidation. Also, oxidative phosphorylation and Pgc1α mRNAs that promotes mitochondrial biogenesis are increased by endurance exercise. Thus, these factors are commonly used as exercise markers. Our results showed that the p-AMPK and p-ACC levels in the gastrocnemius muscle were upregulated and the levels of mRNAs encoding PGC1α, oxidative phosphorylated genes tended to rise; 4 weeks of voluntary wheel exercise was sufficient for the following studies.

EVs consist of a bilayer membrane that makes it possible to travel inter-organs. We isolated EVs from each group's sera for demonstrating the differences between groups. The EVs of both the exercised and sedentary groups 5 μg of EVs contained CD9 which is considered as EVs positive marker. The EVs were characterized *via* nanoparticle tracking. In accordance with the previous result (Oliveira et al., 2018), the EVs diameter, size, and number did not differ between the groups. Recent studies showed that EVs were released during cycling and declined when resting (Frühbeis et al., 2015; Whitham et al., 2018; Brahmer et al., 2019). Another study demonstrated that plyometric jumping and downhill running did not change vesicle number or size (Lovett et al., 2018). Differences in EVs diameter, numbers, and size vary by species, the type and intensity of exercise, and the timing of measurements.

EVs can carry proteins nucleic acids, lipids, and metabolites. Among that, miRNAs are small non-coding RNAs that regulate protein expression *via* mRNA degradation or inhibition of translation. Specific miRNAs are associated with chronic fatigue syndrome, depression, Alzheimer's disease, and Huntington's disease (Hoss, 2015; Wei et al., 2016; Baraniuk and Shivapurkar, 2017; Wu et al., 2017). However, few reports focused on miRNA changes in exercise-induced EVs (D'Souza et al., 2018; Lovett et al., 2018; Hou et al., 2019; Yin et al., 2019; Nair et al., 2020). Therefore, We compared the EVs miRNAs through miRNA sequencing in sedentary and exercised mice; 46 miRNAs were upregulated and 36 downregulated in the exercise group. Among that, miR-10b-5p was upregulated in the rat hippocampus after 4 weeks of treadmill exercise (Fernandes et al., 2018) and miR-let-7i was downregulated in the plasma of athletes after exercise (Nielsen et al., 2014), consistent with our findings. Furthermore, as our results, the elevation of miR-342-5p in exercise-released exosome showed that reduced apoptosis and LDH release (a marker of tissue damage), increased cardiomyocyte viability (Hou et al., 2019). Wang et al. (2019) demonstrated that upregulated exosomal miR-29 plays important role in muscle atrophy and kidney fibrosis in mice. Thus, we confirmed those miRNA sequencing data through RT-qPCR in sera isolated miRNAs (Supplementary Figure 1) and illustrated cumulative distribution plots of miR-29b-3p target and non-target mRNAs (Supplementary Figure 2).

We defined all miRNA-targeted genes with the aid of Target Scan and miRDB, and performed a functional analysis with the aid of Cytoscape. The genes were involved in neurotrophin signaling, central nervous system neuronal development and cerebral cortex cell migration, and peripheral nerve development. Previous studies also confirmed that exercise-induced EVs play roles in nervous system communication (Galieva et al., 2019; Liu et al., 2019). Physical activity modulates the miRNA level which can affect on memory (Fernandes et al., 2017). Also, Exercise-induced extracellular derivatives enhanced neural maturation (Moon et al., 2020). Therefore, we treated Neuro2A cells with exercise-induced EVs 10 μg and it exhibited enhanced viability; the effects of sedentary EVs were less clear. This result indicates that exercise-derived EVs might be actively involved in anxiety disorders. However, because we could not have conducted the miRNA-mediated gene silencing, it might not relevant between miRNAs and cell viability.

A previous study shows that EVs are delivered hippocampus through ICV injection (Nakano et al., 2016; Micci et al., 2019). According to the positive effect on Neuro2A cell viability result of *in vitro* study, the anxiety model was made by 3 weeks of restraint stress and then treated EVs directly through ICV injection. Chronic stress diminishes the neurogenesis (Duman, 2009). Restraint is well-known to induce depressive- and anxiety-like behavior (Chiba et al., 2012; Ampuero et al., 2015). The nest-building test showed that restraint stress evoked anxiety-like behavior. However, tail suspension test demonstrated a tendency in depressive-like behavior. In earlier work, 2 h per day over 7 days of restraint stress-triggered adaptive behavioral changes in anxiety- and depressive-like phenotype (Sadler and Bailey, 2016). Likewise, a previous study demonstrated that differential effects between the restraint methods (Shoji and Miyakawa, 2020).

After ICV injection, exercise-induced EVs treated group was rescued nest-building and central zone entries to significantly greater extents than sedentary. Exercise-derived EVs, thus alleviating anxiety. However, depressive behavior was not affected. We inject only one EV dose; this may be a limitation of the study. In addition, the 3 days of rehabilitation may have diluted the EV effects. Repeat EV injections *via* a cannula or osteomic pump are required in a future study.

We used a microarray to explore changes in hippocampal gene expression. In the restrained group, genes involved in long-term depression (Plcb4, Gucy1a3, Prkg2, Ppp1r17, Grid2, and Crhr1) were downregulated compared to the control group. These genes are also associated with anxiety; medial septal Plcb4 knockout mice exhibited more anxiety-like behaviors (Shin et al., 2009). Gucy1a3 expression in the amygdala correlated positively with social behavior in the rhesus monkey (Sabatini et al., 2007). Prkg2 encodes cGK type II (cGKII), which regulates hippocampal synaptic plasticity (Serulle et al., 2007). Anxiety-like behaviors increased in cGKII knockout mice (Werner et al., 2004). Crhr1 encodes corticotropin-releasing factor 1 (CRF_1_); a CRF_1_ antagonist was anxiolytic in mice (Marcinkiewcz et al., 2016), associated with dose-related changes in regional cerebral glucose metabolism in regions involved in mood and anxiety (Schmidt et al., 2009). Ppp1r17 and Grid2 have not been studied in the context of anxiety.

Exercise-induced EVs injected restrained group exhibited higher levels of the neuroactive ligand-receptor interaction genes (Tacr3, Gabrr1, Cysltr1, Oprm1, and Trhr) than sedentary-derived EVs injected group. Tacr3 overexpression in the unilateral habenula significantly reduced anxiety-like behavior (Cui et al., 2020). By contrast, Tacr3 expression in the amygdala of the NIH-HS rat was higher in a high-anxious group compared with a low-anxious group (Díaz-Morán et al., 2013). Gabrr1 and Cysltr1 has few previous studies related to anxiety and depression. Gabrr1 encodes an ionotropic GABAA receptor that inhibits neuronal activity. Early-life stress such as maternal separation can evoke anxiety-like behavior later in life (Wang et al., 2020). Gabrr1 was upregulated in the pineal gland of rats subjected to long-term maternal separation (Steine et al., 2016). Cysltr1 encodes cysteinyl leukotriene receptor 1; hippocampal knockdown prevented depressive-like behavior in mice (Yu et al., 2016). Oprm1 has been extensively studied in the context of single nucleotide polymorphisms (SNPs) (Mague and Blendy, 2010; Mura et al., 2013; Boparai et al., 2018). The gene encodes the μ opioid receptor. The G allele is a minor form associated with higher resting cortisol levels, blunting of the cortisol response to stress, and separation anxiety disorder (Mague and Blendy, 2010; Boparai et al., 2018). Also, the SNP affects the mRNA expression level, reducing expression by 50% compared to that of the A allele, inducing anxiety (Mura et al., 2013). Trhr encodes the receptor for thyrotropin-releasing hormone (TRH); TRH-R1 knockout mice exhibited increased anxiety- and depressive-like behaviors (Zeng et al., 2007). Low levels of exploratory activity in novel environment rats which are highly inhibited and anxious, depressive-like behavior expressed low Trhr level in dentate gyrus where neural proliferation within the hippocampus (Birt et al., 2021).

We found no significant difference in EV physical properties between the exercise and sedentary groups, but their physiological characteristics varied (in a manner that may affect the nervous system). Exercise-induced EVs contributed to neuronal cell viability and mitigated anxiety-like behavior. Thus, exercise-induced EVs may serve as anxiolytics.

## Data Availability Statement

The datasets presented in this study can be found in online repositories. The names of the repository/repositories and accession number(s) can be found at: https://www.ncbi.nlm.nih.gov/geo/query/acc.cgi?acc=GSE168765.

## Ethics Statement

The animal study was reviewed and approved by Institutional Animal Care and Use Committee of Seoul National University.

## Author Contributions

KY wrote the paper and collected the data. SP contributed data and performed the analysis. SK performed the analysis. HM designed the experiments. All authors contributed to the article and approved the submitted version.

## Conflict of Interest

The authors declare that the research was conducted in the absence of any commercial or financial relationships that could be construed as a potential conflict of interest.

## Supplementary Material

Supplementary Figure 1 The validation of 4 weeks of voluntary wheel exercise mice sera-isolated EVs miRNAs through RT-qPCR. **(A–D)** miR-10b-5p, miR-let-7i-5p, miR-342-3p, and miR-29b-3p miRNA levels normalized by miR-16. ^***^*p* < 0.001, ^****^*p* < 0.0001 vs. S. Statistical analysis was performed with the aid of the one-tailed Student *t*-test. S, sedentary group (*n* = 6); E, exercise group (*n* = 6). The data represent means ± SEM (^*^*p* < 0.05).

Supplementary Figure 2 Cumulative distribution plots of miR-29-3p target and non-target genes. **(A)** Fold change means restraint / non-restraint group. **(B)** Fold change means restraint exercise / restraint sedentary group.
