# Supplementary figures and images for: Effects of Voluntary Running Wheel Exercise-Induced Extracellular Vesicles on Anxiety
Source: Front Mol Neurosci. 2021 Jul 1;14:665800. doi: 10.3389/fnmol.2021.665800 (PMC8280765; doi:10.3389/fnmol.2021.665800)

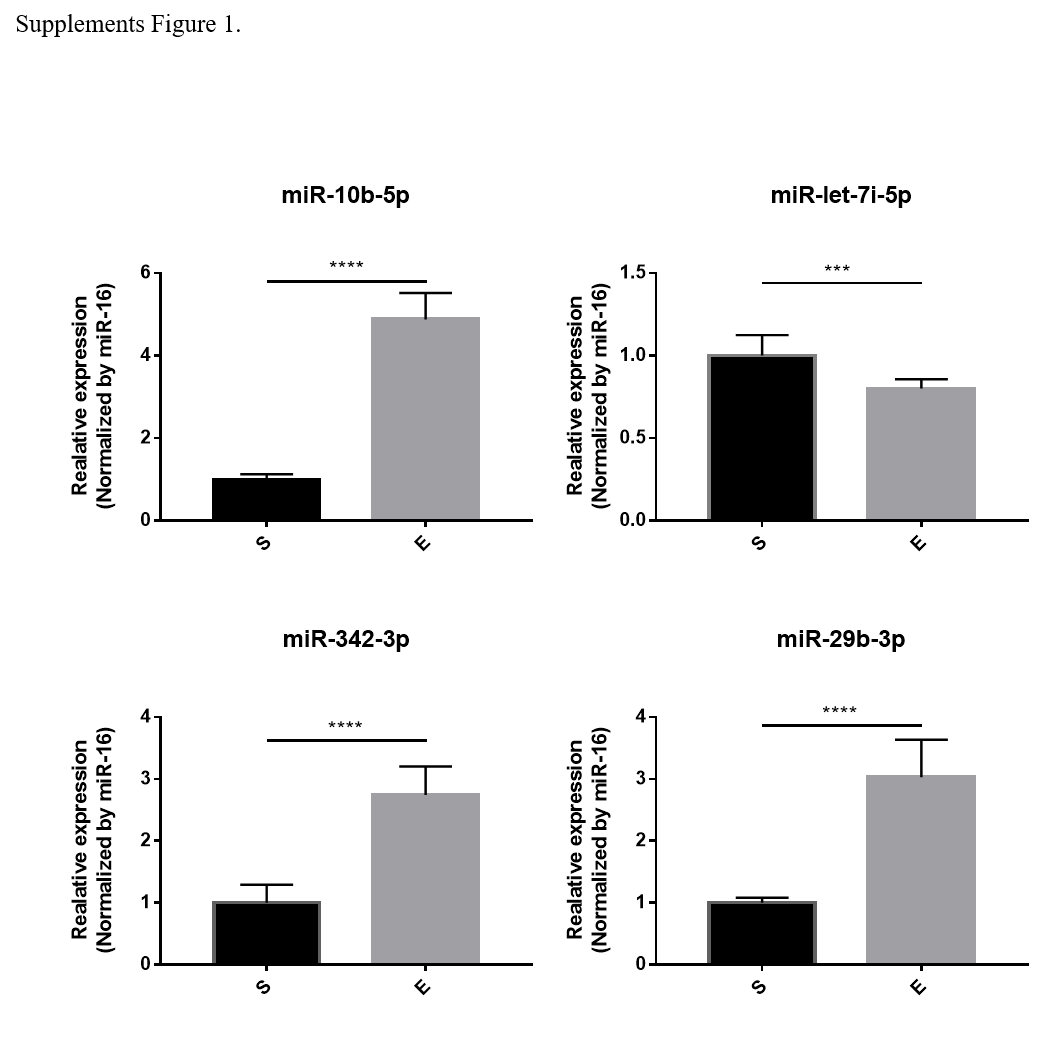

Supplement: Supplementary Figure 1 — The validation of 4 weeks of voluntary wheel exercise mice sera-isolated EVs miRNAs through RT-qPCR. (A–D) miR-10b-5p, miR-let-7i-5p, miR-342-3p, and miR-29b-3p miRNA levels normalized by miR-16. ***p < 0.001, ****p < 0.0001 vs. S. Statistical analysis was performed with the aid of the one-tailed Student t-test. S, sedentary group (n = 6); E, exercise group (n = 6). The data represent means ± SEM (*p < 0.05). [file Image_1.PNG]

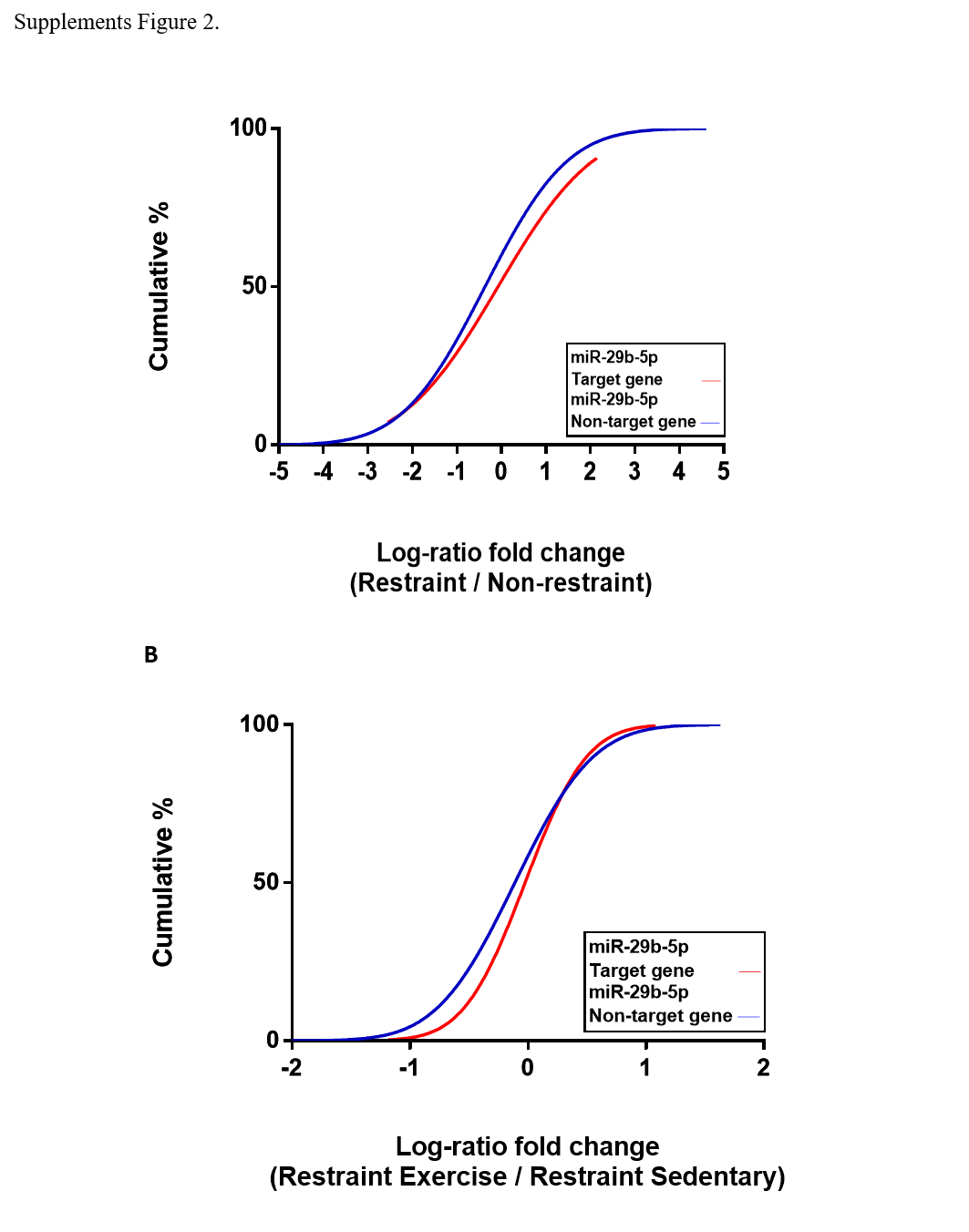

Supplement: Supplementary Figure 2 — Cumulative distribution plots of miR-29-3p target and non-target genes. (A) Fold change means restraint / non-restraint group. (B) Fold change means restraint exercise / restraint sedentary group. [file Image_2.PNG]
